# Human Endogenous Retrovirus K (HML-2) in Health and Disease

**DOI:** 10.3389/fmicb.2020.01690

**Published:** 2020-07-17

**Authors:** Bei Xue, Leonardo A. Sechi, David J. Kelvin

**Affiliations:** ^1^Division of Immunology, Shantou University Medical College, Shantou, China; ^2^Department of Microbiology and Immunology, Canadian Center for Vaccinology, Dalhousie University, Halifax, NS, Canada; ^3^Department of Biomedical Sciences, University of Sassari, Sassari, Italy; ^4^Mediterranean Center for Disease Control, University of Sassari, Sassari, Italy

**Keywords:** HERV-K (HML-2), viral proteins, LTR, polymorphic integration, diseases

## Abstract

Human endogenous retroviruses (HERVs) are derived from exogenous retrovirus infections in the evolution of primates and account for about 8% of the human genome. They were considered as silent passengers within our genomes for a long time, however, reactivation of HERVs has been associated with tumors and autoimmune diseases, especially the HERV-K (HML-2) family, the most recent integration groups with the least number of mutations and the most biologically active to encode functional retroviral proteins and produce retrovirus-like particles. Increasing studies are committed to determining the potential role of HERV-K (HML-2) in pathogenicity. Although there is still no evidence for HERV-K (HML-2) as a direct cause of diseases, aberrant expression profiles of the HERV-K (HML-2) transcripts and their regulatory function to their proximal host-genes were identified in different diseases. In this review, we summarized the advances between HERV-K (HML-2) and diseases to provide basis for further studies on the causal relationship between HERV-K (HML-2) and diseases. We recommended more attention to polymorphic integrated HERV-K (HML-2) loci which could be genetic causative factors and be associated with inter-individual differences in tumorigenesis and autoimmune diseases.

## Introduction

Endogenous retroviruses (ERVs) are the remnants of ancient germ cell infections by exogenous retroviruses that became incorporated into germ line DNA (Gifford and Tristem, 2003). Since the initial infection into their host genome, ERVs have entered into a long-standing union with the rest of the human genome and are inherited in a Mendelian fashion rather than by the horizontal spread typical of an infectious virus. Additionally, ERVs were subjected to natural selection, preventing their expression of some or all proviral genes by accumulating mutations (Jern and Coffin, 2008). As well as accumulating mutations, epigenetic modifications provide a further mechanism for silencing provirus expression (Rowe and Trono, 2011; Leung and Lorincz, 2012). Therefore, ERVs have been regarded as “junk” DNA without biological functions for a long time.

In recent years, accumulated evidence has suggested that not all ERVs have remained silent passengers within our genome but that some have been co-opted into physiological roles in the host. The more direct advantage for the host of endogenization may be the protection against infection by related exogenous viruses (Yan et al., 2009; Hilditch et al., 2011; Blanco-Melo et al., 2017). Moreover, although it is not clear whether the ERV expression that accompanies neuroinflammation is beneficial or detrimental, emerging evidence from studies now support that overexpression of HERV-K in the human brain is not deleterious and may exert neuroprotective effects (Bhat et al., 2014; Manghera et al., 2014, 2015; Mortelmans et al., 2016; Garcia-Montojo et al., 2018; Küry et al., 2018; Dembny et al., 2020). Human ERVs (HERVs) have been proved to be important determinants of pluripotency in human embryonic stem cells and of the reprogramming process of induced pluripotent stem cells (Göke et al., 2015; Grow et al., 2015). HERVs may be involved in embryonic growth. The expression level of HERV-K decreased in preterm newborns, term newborns, infants, and childhood in proper order. A significant inverse correlation found between HERV-K transcripts and duration of pregnancy declares their potential involvement in early life events (Bergallo et al., 2019).

Nevertheless, compared with those possible physiological functions of ERVs, their pathological effects in relation to disease cause more concern. Although infectious counterparts of HERVs have yet to be identified in humans, some exogenous retroviruses related to ERVs demonstrably still exist as infectious agents in some other species; for example, sheep (Palmarini et al., 2004) and cats (Roy-Burman, 1995). HERV-K (HML-2) group, the most recent retroviruses to colonize the human germ line, which is most closely related to the exogenous mouse mammary tumor virus (MMTV) causing breast cancer in mice (Gallahan and Callahan, 1987; Shackleford et al., 1993), has the greatest coding competence and is acknowledged to be the most biologically active class of HERVs (Bannert and Kurth, 2004). Current research indicates that reactivation of HERV-K (HML-2) potentially contributes to disease pathogenesis, such as ovarian cancer (Wang-Johanning et al., 2007), prostate cancer (Wang-Johanning et al., 2003a), melanoma (Büscher et al., 2005), rheumatoid arthritis (RA) (Freimanis et al., 2010), systemic lupus erythematosus (SLE) (Balada et al., 2009), as well as multiple sclerosis (MS) (Perron and Lang, 2010). As the expression of HERV-K (HML-2) is also detected in normal tissue types, which indicates their biological functions in physiological activities (Ehlhardt et al., 2006; Schmitt et al., 2015), whether HERV-K (HML-2) is an initiator of diseases or is simply an ancillary consequence of transformation calls for further discussion (Young et al., 2013). In this review, we summarized the advances between HERVs and diseases to provide basis for further studies on the relevance between HERV-K (HML-2) and diseases.

## Structure of HERV-K (HML-2)

ERVs are commonly grouped into three different classes because of their phylogenetic similarity to exogenous retroviruses. The structure of HERV-K (K denoting a lysine tRNA primer binding site) elements resembles a typical beta-retrovirus (Jern et al., 2005). Within the HERV-K family, there are several subgroups denoted HML-1 to HML-11 (human endogenous MMTV-like), and each subgroup can be traced back to distinct germ-line infections by exogenous retroviruses (Subramanian et al., 2011). Members of the HML-2 subgroup, which has been active and infectious for much of the past 30 million years, are suggested to be the most recent integrations. Many members were inserted into the human genome after the divergence of humans and chimpanzees approximately 6 million years ago (Reus et al., 2001; Subramanian et al., 2011). Although mutational events have rendered most of the HML-2 replication defective following integration, the HML-2 group is acknowledged to be the most biologically active subgroup of HERV-K with many of them still transcriptionally active (Wallace et al., 2014) and able to retain the ability to encode functional retroviral proteins (Muster et al., 2003; Büscher et al., 2005) and produce retrovirus-like particles (Contreras-Galindo et al., 2008).

A full-length HERV element is approximately 9.5 kb in length and consists of an internal region of four essential viral genes (*gag*, *pro*, *pol*, and *env*) and two long terminal repeats (LTRs). The *gag* gene encodes core structural proteins encoding group-specific antigens such as capsid and nucleocapsid proteins. The *pro* gene encodes a protease and the deoxyuridine triphosphate nucleotidohydrolase (dUTPase) and can be found within the N-terminal region (Hohn et al., 2013). The *pol* gene encodes viral enzymes, in particular, reverse transcriptase, the enzyme responsible for viral DNA synthesis using the viral RNA genome. The *env* gene encodes the viral envelope glycoprotein that is important for receptor recognition and membrane fusion. The *env* gene helps in the classification of HERVs from other LTR retrotransposons (Löwer et al., 1996; Griffiths, 2001; Khodosevich et al., 2002; Prudhomme et al., 2005; Balada et al., 2009). The HERV-K (HML-2) proviruses can be classified into two sub-types according to the presence or absence of a 292 bp “deletion” at the pol-env boundary. Type II proviruses express the accessory Rec protein, which resembles the HIV Rev and HTLV Rex proteins, and mediates transport of unspliced or partially spliced HERV-K (HML-2) mRNAs from the nucleus into the cytoplasm (Magin et al., 1999; Macfarlane and Simmonds, 2004). Type I proviruses have a 292 bp deletion that prevents expression of the Rec protein but can instead express an alternative protein, Np9, which has no known physiological function in HERV-K (HML-2) replication. The identical LTRs located at both the ends of HERVs contain many regulatory elements such as promoters, enhancers, and polyadenylation signals required for retroviral gene expression (Buzdin et al., 2003; Dunn et al., 2005). Additionally, LTRs can have significant effects on neighboring host gene expression (Jern and Coffin, 2008) and contribute to the genome-wide distribution of regulatory sequences that allows cells to respond to a single stimulus by regulating a series of its genes (Savage et al., 2019).

## Human-Specific and Polymorphism Integration of HERV-K (HML-2)

**FIGURE 1:**
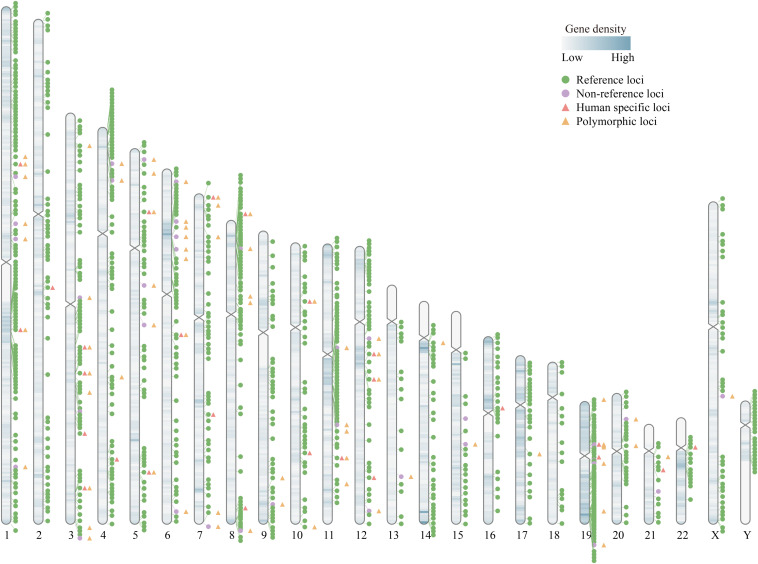
Diagram of the genome wide distribution of HERV-K (HML-2). There are 1098 HERV-K (HML-2) loci in human genome, including reference (hg19) and non-reference loci. Among them, there are 26 reported human-specific loci and 71 polymorphic integration loci, which have been highlighted with different symbols in the figure (except one polymorphic locus, solo-LTR946, located in GL000219.1). Detailed information of all the 1098 HERV-K (HML-2) loci were summarized in Xue et al. (2020).

It is known that there are more than 1,000 HERV-K (HML-2) loci in the human genome. Most of them reside in the genome as solo-LTRs generated by homologous recombination between the LTRs of a single HERV-K (HML-2), resulting in the deletion of the internal sequence (Hughes and Coffin, 2004). Solo-LTRs are approximately 10-fold more abundant than their full-length or nearly full-length proviral integrations (Subramanian et al., 2011). In total, there are 1098 reported loci whose insertional elements possess functional elements, including 1061 reference (hg19) insertions and 37 non-reference insertions (Subramanian et al., 2011; Lee et al., 2012; Marchi et al., 2014; Wildschutte et al., 2016; Wallace et al., 2018; Xue et al., 2020). The genome wide distribution of all 1098 loci is shown in Figure 1 using RIdeogram (Hao et al., 2020).

As the most recently integrated family of HERV-K, HML-2 is the only group which comprises a number of evolutionarily young proviruses that are human-specific with multiple HML-2 members appearing polymorphic in the human population (Jha et al., 2011). Human-specific HERV-K (HML-2) elements have integrated into the human genome since the divergence of humans and chimpanzees. In recent years, the number of known human-specific HERV-K (HML-2) has risen and the absence of these loci in our closest primate relatives, chimpanzees, indicates that these elements may have contributed to the genomic divergence between humans and chimpanzees by regulating the expression of cellular genes and mediating chromosome rearrangements (Figure 1; Turner et al., 2001; Macfarlane and Simmonds, 2004; Subramanian et al., 2011). Insertionally polymorphic HERV-K (HML-2) loci may have different structures, including full-length proviral integrations, solo-LTR integrations, and unoccupied pre-integration in different individuals (Hughes and Coffin, 2004; Moyes et al., 2007). Seventy-one polymorphic HERV-K (HML-2) integrations have been identified in different ethnic and case-control cohort studies (Figure 1; Subramanian et al., 2011; Marchi et al., 2014; Wildschutte et al., 2016; Thomas et al., 2018; Wallace et al., 2018; Xue et al., 2020). Researchers have considered that the existence of polymorphisms may provide one explanation of how ubiquitous elements such as HERV-K (HML-2) can cause disease in only a proportion of individuals (Moyes et al., 2007). Some loci show both polymorphisms and human-specific loci, such as HERV-K113 and HERV-K115. These loci could be involved in human evolution as well as disease modifying, which may contribute to the sensitivity of different ethnic groups to different diseases (Moyes et al., 2005; Marchi et al., 2014). Nowadays, these interesting loci of HERV-K (HML-2) are gaining attention from researchers as a new genomic avenues aid in the study of complex diseases, including cancers and autoimmune diseases.

## HERV-K (HML-2) Act as Potential Causative of Diseases

**FIGURE 2:**
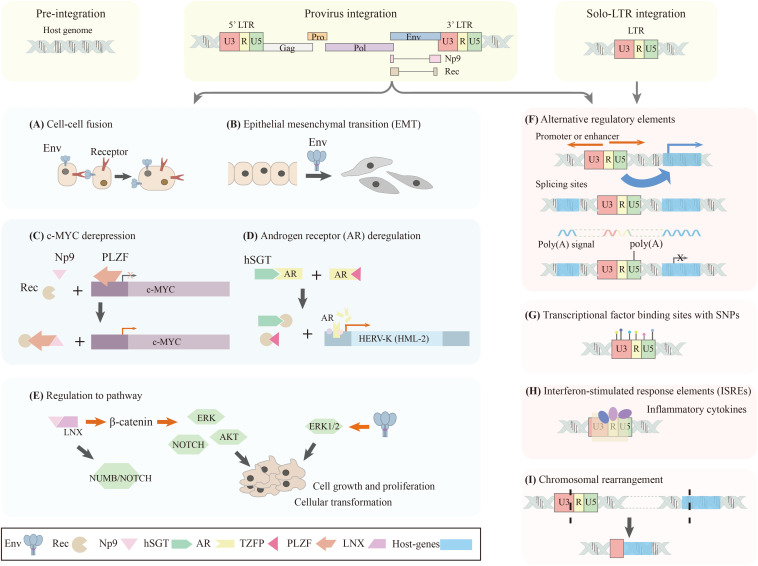
Schematic diagram of potential pathogenetic mechanisms of HERV-K (HML-2). The polymorphic integrated HERV-K (HML-2) locus can have three different status in different individuals, including: (i) Pre-integration: without both viral protein coding sequences and LTRs; (ii) Provirus integration: possesses both viral protein coding sequences and LTRs; (iii) Solo-LTR integration: only possesses an LTR sequence. The presence or absence of viral proteins coding sequences or LTRs may reveal different pathogenetic mechanisms: **(A–E)** show viral proteins produced by HERV-K (HML-2) cause aberrant biological phenomenon; **(F–I)** show regulatory functions of LTRs. **(A,B)** Env protein induces cell-cell fusion and epithelial mesenchymal transition (EMT) which could contribute to tumorigenesis. **(C,D)** Accessory proteins, Rec and Np9, are suggested as oncogenic proteins causing *c-myc* depression and androgen receptor deregulation. **(E)** Viral proteins, such as Env and Np9, can regulate different signal pathways related to cell growth and proliferation. **(F)** LTRs can significantly affect their proximal host genes in addition to regulating viral gene expression because of the possession of regulatory elements. LTRs can act as alternative promoters, enhancers, splicing sites, and poly **(A)** signals. **(G)** LTR sequence variation, causing transcription factor binding site polymorphism, induce different promoter expression patterns. **(H)** The presence of interferon-stimulated response elements (ISREs) in the promoter region of HERV-K (HML-2) suggests its expression could be regulated by inflammatory cytokines. **(I)** LTR-derived chromosomal re-arrangement can induce aberrant expression profile of host genes. Polymorphic integration, a same HERV-K (HML-2) locus showing different status in different individuals, may causing different expression profile of both the viral proteins and their proximal host genes, can be a possible avenue to study the pathogenic functions of HERV-K (HML-2).

An increasing number of studies are now focusing on the pathogenetic mechanisms of HERV-K (HML-2). There are two primary potential mechanisms under investigation (Figure 2). One is the possibility that a viral protein encoded by HERV-K (HML-2), may function as an onco-proteins or induce autoantibodies in the host. The other is the regulatory functions of LTRs or HERV-K (HML-2) loci, which may induce the dysregulation of the host genome, or recombination involving HERV-K (HML-2) sequences might lead to chromosomal instability and large-scale chromosomal anomalies.

### HERV-K (HML-2) Proteins

Both the proviral proteins (Env) and the accessory proteins (Rec, Np9) are suspected to have an etiological role in diseases. The Env protein, the viral envelope glycoprotein, is important for membrane fusion of viruses. It has been reported to induce cell-cell fusion in melanoma which could contribute to tumorigenesis (Huang et al., 2013; Figure 2A). The expression of Env triggers innate and adaptive immunity because of its similarity to exogenous viral protein (Trela et al., 2016). Moreover, the Env protein is a molecular mimic of the oxygen responsive element binding protein, a human nuclear factor that controls the expression of glutathione peroxidase, resulting in reduced production of the enzyme. This further increases the toxicity of free radicals that are no longer under proper control and enhances the risk of cancer (Krone et al., 2005). In breast cancer cells, artificial regulation of *env* expression can affect expression of tumor-associated genes, cell proliferation, migration, and invasion, indicating that Env protein plays an important role in tumorigenesis and metastasis of breast cancer (Zhou et al., 2016). In 293T cells, Env was shown to effectively activate the ERK1/2 pathway, which is associated with cellular transformation. Furthermore, expression of *env* in a non-tumorigenic human breast epithelial cell line induces epithelial to mesenchymal transition (EMT), a crucial event in tumor aggressiveness and metastasis (Lemaître et al., 2017; Figure 2B). Transgenic animals expressing the *env* gene developed progressive motor dysfunction accompanied by selective loss of volume of the motor cortex, decreased synaptic activity in pyramidal neurons, dendritic spine abnormalities, nucleolar dysfunction, and DNA damage (Li et al., 2015). In a murine model system, Env was suspected to be a useful target for vaccine development for diverse types of cancer (Kraus et al., 2013).

The accessory proteins, Rec and Np9, may be oncogenic proteins because of their known interaction with disease-related proteins. The promyelocytic leukemia zinc finger (PLZF) protein, a transcriptional repressor of the *c-myc* proto-oncogene, which can lead to the depression of *c-myc*, interacts with Rec and Np9 (Denne et al., 2007; Figure 2C). In addition, Rec has also been shown to interact with the testicular zinc-finger protein (TZFP) and the human small glutamine-rich tetratricopeptide repeat protein (hSGT), transcriptional repressors of the androgen receptor, which may lead to tumor progression (Kaufmann et al., 2010; Hanke et al., 2013; Figure 2D). There is a putative mechanism for Rec to induce diseases by directly binding to a subset of cellular RNAs and to modulate their ribosome occupancy with unknown cellular consequences (Grow et al., 2015). Np9 interacts with Ligand of Numb protein X (LNX), an E3 ubiquitin ligase that binds members of the NUMB/NOTCH pathway (Armbruester et al., 2004), and has also been reported to upregulate β-catenin, to activate ERK, AKT and NOTCH 1 pathways, to promote the growth of human leukemia stem cells (Chen et al., 2013; Figure 2E). *np9* and *rec* expression are significantly correlated with a range of cancer stem cell and EMT biomarkers, including cellular receptors, transcription factors, and histone modifiers, suggesting that HERVs may be good candidate biomarkers in identifying the transitional cancer stem cell-like states that are present during the progression of EMT and cancer metastasis (Lin et al., 2018). Np9 was suspected to control viability and migration of teratocarcinoma cells which support the implication that Np9 has oncogenic potential (Chan et al., 2019).

Other viral proteins, such as the protease encoded by Pro, are also potential candidates in causing diseases. A recent paper claimed that hundreds of cellular proteins are potential substrates of the HERV-K (HML-2) protease (Pro). This has received little attention so far, suggesting that even low-level expression of HERV-K (HML-2) Pro may affect a diverse array of proteins, thus having a functional impact on cell biology and possible relevance for human diseases (Rigogliuso et al., 2019). In pulmonary arterial hypertension, the HERV-K dUTPase was shown to activate B cells, elevate cytokines in monocytes and pulmonary arterial endothelial cells, and increase pulmonary artery vulnerability to apoptosis, thus contributing to sustained inflammation, immune dysregulation, and progressive obliterative vascular remodeling (Saito et al., 2017). Human primary cells treated with wild-type and mutant HERV-K dUTPase proteins triggered the secretion of TH1 and TH17 cytokines involved in the formation of psoriatic plaques, including IL-12p40, IL-23, IL-17, TNF-α, IL-8, and CCL-20, which support dUTPase as a potential contributor to psoriasis pathophysiology (Ariza and Williams, 2011).

### Long Terminal Repeats (LTRs)

LTRs, containing many regulatory elements, can have significant effects on their proximal host genes in addition to regulating retroviral gene expression. Due to the large numbers of the LTR elements compared to proviral integrations, and their possession of numerous of regulatory elements, the regulatory functions of LTRs are worthy of attention. Although LTRs are usually suppressed by epigenetic factors, it is estimated that retroviral LTRs initiate 10 times as many transcripts as regular promoters even in normal healthy cells (Conley et al., 2008a, b; Cohen et al., 2009; Gogvadze et al., 2009). Activation/silencing of LTR5_Hs was suggested to be associated with reciprocal up- and down-regulation of hundreds of human genes (Fuentes et al., 2018; Figure 2F). Sometimes, LTRs activity could have a beneficial effect, such as driving the expression of tumor suppressor genes which are commonly silenced in tumors (Kassiotis and Stoye, 2017). Two HERV-K (HML-2) loci can participate in the specific antisense regulation of human gene expression, suggesting their involvement in the evolutionary process (Gogvadze et al., 2009). The majority of studies, however, are linked to deleterious effects. Global hypomethylation, including LTR hypomethylation, is one of the common consequences of tumorigenesis, which will result in promoter activation that might induce more initiations (Reiss et al., 2007; Kreimer et al., 2013). An *in vitro* model of human mammary epithelial cell transformation indicated the 5′LTR promoter activity is active in tumorigenic cells only, suggesting that the cellular environment of a cancer cell is a critical component for induction of LTR promoter activity (Montesion et al., 2017). LTR sequence variations, causing transcription factor binding site polymorphisms, are positively correlated with promoter expression patterns in human breast cancer cell lines (Montesion et al., 2018; Figure 2G). The presence of interferon-stimulated response elements (ISREs) in the promoter region of HERV-K suggests its expression could be regulated by inflammatory cytokines, which could explain its implication in autoimmune disorders (Manghera et al., 2016; Figure 2H). A HERV-K (HML-2) locus in 22q11.21 acts as a tissue-specific enhancer for the PRODH gene, which is one of the candidate genes for susceptibility to schizophrenia and other neurological disorders (Maria et al., 2013). Therefore, it is suspected that LTRs are involved in different diseases functioning as regulatory elements. In addition, the role of HERV-K (HML-2) in chromosomal rearrangement, resulting from non-allelic recombination, has been demonstrated. In prostate cancer, the rearrangement involves the translocation of HERV-K (22q11.23) 5′LTR upstream and the transcription factor ETS, that results in overexpression of the oncogene ETV1 (Tomlins et al., 2007; Figure 2I).

### Polymorphic Integration

Insertionally polymorphic HERV-K (HML-2) integrations can affect both viral protein production and host-gene regulation, further supporting the idea that specific HERV-K (HML-2) signatures may be associated with the potential pathogenicity of HERV-K (HML-2) in different individuals. A recent study pointed out that HERV-K polymorphisms can be associated with neurologic and immunologic disease phenotypes based on a phenotype-wide scan (Wallace et al., 2018). Some specific polymorphic HERV-K (HML-2) loci have been documented as genetic risk factors in some diseases (Table 1). To date, reports predominantly focus on HERV-K113 and HERV-K115, as they are the most recently integrated and conserved HERV-K (HML-2) loci known. In the United Kingdom, HERV-K113 was found to be significantly increased in the prevalence in Sjogren’s syndrome and MS patients, although there was no increased prevalence of HERV-K115 in either disease (Moyes et al., 2005). The prevalence of insertionally polymorphic HERV-K113 was significantly increased in Polish patients with SLE and RA (Krzysztalowska-Wawrzyniak et al., 2011). There is also additional research on other polymorphic HERV-K (HML-2) loci. An insertionally polymorphic solo LTR (1p13.2) is suggested to be involved with the susceptibility of lung adenocarcinoma in female never-smokers in an age-dependent manner (Kahyo et al., 2013). A paper declared that a polymorphic locus, in 5q14.1, integrated within RASGRF2, can disrupt host gene transcription and is associated with drug addiction (Karamitros et al., 2018). In some studies, there were no correlations between polymorphic loci frequency and diseases. In breast cancer patients and an equal number of age-matched controls with no history of malignancy, HERV-K113 and HERV-K115 showed no significant difference in frequency and no apparent association with other clinical information (Burmeister et al., 2004). In another case-control analysis investigating the possible relationship between the presence of polymorphic HERV-K (HML-2) proviruses with the occurrence of breast cancer, 10 additional proviral loci showed no significant differences in the prevalence between groups (Wildschutte et al., 2014). In Japanese schizophrenia patients and healthy controls, researchers found no difference in the frequency of HERV-K115, which suggested that HERV-K115 may not be associated with schizophrenia in general, but they found that HERV-K115 could play a partial role in early precipitation of the disease (Otowa et al., 2006). To date, results on the relevance between polymorphic HERV-K (HML-2) integrations and diseases appears mixed, as some studies show significant increases in frequency in patients, but others do not (Table 1). There are two potential reasons. One reason is that all the studies based on specific PCR results were performed only in about 100 samples, which resulted in the positive or negative results restricted to the number of samples. The other reason is that most of the studies focused on only a few special loci, especially HERV-K113 and HERV-K115. Following integration, HERV-K (HML-2) and the host genome proceed into co-option and a provirus with negative effects will certainly be removed from the population by selection and have a reduced probability of population fixation. Therefore, those HERV-K (HML-2) loci with rare frequencies among humans would be more appropriate candidates for inference of disease-associated loci (Wildschutte et al., 2014). Within the above limited studies, all the polymorphic provirus integrations related to diseases were concentrated in autoimmune diseases; while there was no cancer studies. Polymorphic integrations involved in cancer were solo-LTR integrations. However, investigations are still needed to identify and verify the involvement of polymorphic HERV-K (HML-2) integrations and their molecular mechanisms in specific diseases.

**TABLE 1:** Polymorphic integrated HERV-K (HML-2) and diseases.

**Locus**	**Coordinate in hg19**	Alleles*	**Relevance to diseases**	**References**
HERV-K113	Chr19: 21841535–21841541	Pro/pre	Higher frequency in Sjogren’s syndrome, MS, SLE, and RA	Moyes et al., 2005; Krzysztalowska-Wawrzyniak et al., 2011
1p13.2	Chr1: 111802591–111802597	Solo/pre	Higher frequency in Lung adenocarcinoma	Kahyo et al., 2013
5q14.1	Chr5: 80442265–80442271	Solo/pre	Higher frequency among individuals with strong addictive behavior, LTR insertion can modulate RASGRF2 transcription	Karamitros et al., 2018
HERV-K115	Chr8: 7355396–7364859	Pro/pre	No significant increase frequency in breast cancer, schizophrenia, Sjogren’s syndrome, and MS patients	Burmeister et al., 2004; Moyes et al., 2005; Otowa et al., 2006
HERV-K113	Chr19: 21841535–21841541	Pro/pre	No significant difference in the prevalence of	Burmeister et al., 2004
1p31.1	Chr1: 75842770–75849143	Pro/pre	proviruses between breast cancer patients	Wildschutte et al., 2014
3q13.2	Chr3; 112743478–112752282	Pro/solo/pre	and controls	
6q14.1	Chr6: 78427018–78436083	Pro/solo		
7p22.1	Chr7: 4630560–4640031	Pro/solo/pre/tandem		
8p23.1	Chr8: 8054699–8055725	Pro/pre		
10p12.1	Chr10: 27182398–27183380	Pro/solo		
11q22.1	Chr11: 101565793–101575259	Pro/solo/pre		
12q13.2	Chr12: 55727213–55728183	Pro/solo/pre		
12q14.1	Chr12: 58721241–58730698	Pro/solo		
19p12	Chr19:21841535–21841541	Pro/pre		

*pro, provirus integration; solo, solo-LTR integration; pre, pre-integration.

### *De novo* Integration

Similar to integration polymorphisms, *de novo* integration of HERV-K (HML-2) is another possible mechanism inducing diseases. Although none of the currently known HERV-K (HML-2) elements appear to have the capacity to produce infectious particles and be actively transposed in human genome, the existence of human-specific and polymorphic integration loci support the notion of infectious particle activity for much of the past 30 million years, especially insertions into the genome after the divergence of humans and chimpanzees approximately 6 million years ago. Although there is no evidence of *de novo* chromosomal integration, modern HERV-K (HML-2) viruses have been documented to retain the ability to be reverse transcribed and transmitted to other cells (Contreras-Galindo et al., 2015). The existence of an infectious HERV-K (HML-2) provirus with low prevalence in the current human population cannot be completely ruled out (Hohn et al., 2013). To better understand the relevance between polymorphic HERV-K (HML-2) integrations and diseases, and whether there is *de novo* integration or not, an efficient method for identifying genome-wide distribution of HERV-K (HML-2) loci in the human is urgently needed. For this purpose, we developed PCR-based target enrichment sequencing of HERV-K (HML-2) to assess the genome-wide distribution of HERV-K (HML-2) in the human genome (Xue et al., 2020).

## Aberrant Behavior of HERV-K (HML-2) as Epiphenomenon of Diseases

Although the functional associations have not been completely clarified, a growing number of reports continue to demonstrate increased aberrant behavior of HERV-K (HML-2) in several types of human diseases. The aberrant behavior included, but was not limited to, the abnormal expression of viral proteins, antibodies of viral proteins, and viral-like particles.

### Expression of HERV-K (HML-2) Transcripts

One of the most common phenomenon associated with HERV-K insertions is the aberrant expression of viral transcripts, which were suggested as diagnostic or prognostic markers in some diseases. As it is closely related to the exogenous MMTV causing breast cancer in mice, HERV-K (HML-2) has drawn attention to its possible role in breast cancer. The expression of HERV-K *env* transcripts was observed to be significantly higher in breast cancer tissues than in normal breast tissue (Wang-Johanning et al., 2003b), and its expression with or without therapy suggests the potential as a diagnostic marker for breast cancer (Rhyu et al., 2014). Moreover, high expression of HERV-K Env protein in breast cancer patients is associated with markers of disease progression and poor disease outcome, indicating that Env could possibility be used as a novel candidate prognostic marker for breast cancer (Zhao et al., 2011). Significantly increased expression of HERV-K108, HERV-K109, HERV-K113, and HERV-K115 in the basal breast cancer subtype compared to other breast cancer subtypes indicates the possible association of HERV-K expression with extremely poor prognosis, high frequencies of recurrence and metastasis, suggesting these loci as possible targets for cancer vaccines or immunotherapy (Johanning et al., 2017). Reverse transcriptase protein expressed in early malignancy might also serve as a novel prognostic marker for breast cancer (Golan et al., 2008). There is also evidence of abnormal expression in other cancers. The significantly different expression of HERV-K (12q14.1) *env* mRNA among different types of lung cancer and healthy controls indicates the potential of HERV-K as promising non-invasive blood-based biomarker for prognosis, early detection, and monitoring of lung cancer (Zare et al., 2018). Upregulation of HERV-K (HML-2) in hepatocellular carcinoma patients was significantly related to cancer progression and poor outcome, indicating that HERV-K (HML-2) might be a novel candidate prognostic biomarker for hepatocellular carcinoma (Ma et al., 2016). Upregulation of HERV-K *gag* and *env* expression in blood is possibly an early detection biomarker for prostate cancer (Wallace et al., 2014; Sayanjali, 2017). Additionally, activation of HERV-K expression in melanoma cells is required for the transition toward a more malignant phenotype (Serafino et al., 2009). The HERV-K10 overexpression in leukemia samples might be specifically associated with tumor development (Depil et al., 2002). Significantly high expression observed in acute myeloid leukemia (Januszkiewicz-Lewandowska et al., 2013), acute lymphoblastic leukemia (Bergallo et al., 2017), and chronic lymphocytic leukemia (Fischer et al., 2014), strongly suggests a possible contribution of this gene in the pathogenesis of leukemia. The increased expression of HERV-K in tumors is associated with soft tissue sarcoma (STS) patients’ prognosis (Giebler et al., 2018). Enhanced expressions of HERV-K (HML-2) were detected in melanoma patients (Büscher et al., 2005) and ovarian epithelial tumors (Wang-Johanning et al., 2007).

The abnormal expressions of viral transcripts was also detected in autoimmune diseases and some other diseases. Increased expression of HERV-K *gag*, *pol*, and *env* transcripts were detected in amyotrophic lateral sclerosis (ALS) brain tissue (Douville et al., 2011; Li et al., 2015). Significant increases in HERV-K (HML-2) *gag* activity were seen in RA patients compared to disease controls (Freimanis et al., 2010). HERV-K18 expression was significantly elevated in peripheral blood from patients with juvenile rheumatoid arthritis compared to controls (Sicat et al., 2005). High expression of HERV-K (HML-2), especially HERV-K type 1, can be detected in plasma samples from RA patients (Reynier et al., 2009). The *env* mRNA expression level of HERV-K (HML-2) was significantly upregulated in pemphigus vulgaris patients in comparison to healthy controls, suggesting HERV-K (HML-2) expression could be measured as a possible diagnostic tool for detection of pemphigus vulgaris and monitoring of the treatment (Karimi et al., 2019). HERV-K (HML-2) was significantly overrepresented in bipolar-disorder and schizophrenia-associated samples compared to healthy brains, suggesting a potential association with disease (Frank et al., 2005). In the blood samples from children with specific language impairment, the expression of HERV-K (HML-2) *gag* was at a lower level in comparison with that in control group (Minchev et al., 2019).

Even though abnormal expression of HERV-K groups in diseases has been relatively well studied, little is known about the transcription of specific HERV-K (HML-2) loci. The abnormal expression of specific HERV-K (HML-2) in diseases has been gaining attention recently (Table 2). In melanoma, researchers found that transcription profiles of loci differed significantly between samples. Transcriptional regulation of HERVs appear to be complex requiring specific studies to elucidate which HERV loci are transcribed, and how transcribed HERVs may be involved in disease (Schmitt et al., 2013). With the high-throughput capabilities of next-generation sequencing, HERV-K (HML-2) expression at the level of individual proviruses and secreted virions in the teratocarcinoma cell line Tera-1 were profiled to understand the expression patterns, with transcripts emanating primarily from two proviruses located on chromosome 22 (Bhardwaj et al., 2015). There are some specific HERV-K (HML-2) loci linked to specific diseases. A significant association between HERV-K18 Env genotype and MS risk has been reported, indicating that risk of MS was threefold higher among HERV-K18.3 individuals compared with HERV-K18.2 individuals (Tai et al., 2008). In prostate cancer, the upregulated expression of HERV-K (HML-2) was limited to activation of a few loci, especially 22q11.23 and 3q12.3 (Goering et al., 2011, 2015). Preferential expression of Gag-HERV-K 22q11.23 in prostate cancer tissue and increased frequency of autoantibodies observed in patients with advanced prostate cancer make this protein a potential biomarker to detect the progression and biochemical recurrence rate of prostate cancer (Reis et al., 2013).

**TABLE 2:** Disease-related specific HERV-K (HML-2) loci.

**Locus**	**Coordinate in hg19**	**Characterization**	**Disease**	**References**
3q12.3	Chr3: 101410736–101419859	Frequently upregulated expression	Prostate cancer	Goering et al., 2015
22q11.21	Chr22: 18926186–18935307	Preference transcription in Tera-1	Teratocarcinoma	Bhardwaj et al., 2015
		Tissue-specific enhancer of PRODH	Schizophrenia and other neurological disorders	Maria et al., 2013
22q11.23	Chr22: 23879929–23888810	Chromosome rearrangement inducing overexpression of ETV1	Prostate cancer	Tomlins et al., 2007
		More frequently in detecting autoantibodies to Gag		Reis et al., 2013
		Frequently upregulated expression		Goering et al., 2011, 2015
		Preference transcription in Tera-1	Teratocarcinoma	Bhardwaj et al., 2015
HERV-K10	Chr5: 156084716–156093896	Detection of antibody to HERV-K10 Gag	Rheumatoid arthritis	Nelson et al., 2014
HERV-K18	Chr1: 160660574–160669806	High expression	Juvenile rheumatoid arthritis	Sicat et al., 2005
		Genotype K18.3 showing a high risk of MS	Multiple sclerosis	Tai et al., 2008
HERV-K111	Pericentromeric regions	Deletion of 5′LTR-gag sequence	Cutaneous T-cell lymphoma and Sezary syndrome	Kaplan et al., 2019

### Humoral and Cellular Immune Response to HERV-K (HML-2) Proteins

In addition to the mRNA, humoral and cellular immune response to HERV-K (HML-2) proteins were also observed in various studies. Elevated HERV-K (HML-2) antibodies were found in the blood from early stage breast cancer patients, and were further increased in patients who are at risk of developing a metastatic disease (Wang-Johanning et al., 2014). Furthermore, monoclonal antibodies against HERV-K Env protein, which can inhibit growth and induce apoptosis of breast cancer cells, significantly reduced growth of xenograft tumors in mice, showing potential as novel immunotherapeutic agents for breast cancer therapy (Wang-Johanning et al., 2012). In melanoma, anti-HERV-K Gag and Env antibodies were negatively related to disease-specific overall survival, suggesting that humoral anti-HERV-K immune response may provide additional prognostic information to that of established melanoma markers (Hahn et al., 2008). Autoantibodies against HERV-K Gag together with ERG and AMACR may be a useful panel of biomarkers for diagnosis and prognosis of prostate cancer (Rastogi et al., 2016). Anti-HERV-K antibodies were detected in patients with ovarian cancer, but not in normal female controls, documenting that HERV Env proteins, especially those expressed on the cell surface, may serve as novel tumor targets for detection, diagnosis, and immunotherapy of ovarian cancer (Wang-Johanning et al., 2007). A strong association of HERV-K (HML-2) antibodies and the clinical manifestation of the disease and therapy success suggests that HERV-K (HML-2) antibodies seem to have an important diagnostic value as well as an indicator of chemotherapy success in germ cell tumors (Kleiman et al., 2004). The HERV-K Env-su_19__–__37_ antibodies significantly correlated with clinical measures of disease severity, both in serum and cerebrospinal fluid, suggesting that increased circulating levels of the antibodies could serve as a possible early novel biomarker in patients with ALS (Arru et al., 2018). The autoantibodies against HERV-K (Env-su_19__–__37_) were significantly higher in RA than in healthy controls (Mameli et al., 2017; Bo et al., 2018). A significantly elevated IgG antibody response to an HERV-K10 Gag matrix peptide was observed in patients with RA, suggesting that the exposure of HERV-K10 may cause a secondary, antigenic driven immune response in RA (Nelson et al., 2014).

Moreover, HERV-K proteins can also provide antigenic epitopes for recognition by T cells and B cells. The recombinant transmembrane envelope protein of HERV-K can inhibit the proliferation of human immune cells and modulate the release of cytokines which may suppress immune responses and thus prevent rejection of the tumor (Morozov et al., 2013). The presence of elevated levels of HERV-K Env-specific T-cell proliferation, cytokine production, and HERV-K-specific cytotoxic T lymphocyte activity were identified in breast cancer and ovarian cancer patients (Wang-Johanning et al., 2008; Rycaj et al., 2015). Therefore, researchers believe that HERV-K proteins may represent potential tumor-associated antigens as targets for therapeutic vaccines to these diseases. In human immunodeficiency virus (HIV)-1-positive individuals, specific T cell responses to HERV-K (HML-2) epitopes were identified, and these cell clones can eliminate HIV-1 infected cells *in vitro* (Garrison et al., 2007; SenGupta et al., 2011; Jones et al., 2012). An inverse correlation between anti-HERV T cell responses and HIV-1 plasma viral load demonstrates a role for these T cell responses in helping to contain HIV-1 viremia (Garrison et al., 2007). In addition, anti-HERV-K (HML-2) transmembrane and surface unit protein specific B-cells were also identified in HIV-1 positive patients (Michaud et al., 2014). HERV-K (HML-2) proteins may be a new therapeutic target in HIV-1 infection.

### HERV-K (HML-2) Viral-Like Particles

Although no infectious exogenous retrovirus has been documented to date, HERV-K viral-like particles that were first found to be produced in teratocarcinoma cell lines (Boller et al., 1993; Ruprecht et al., 2008) have been observed in other disease types. Three different types of retrovirus-like particles, different in the presence of viral surface proteins and the existence of free mature virions, were observed in human teratocarcinoma cell lines, suggesting that different HERV-K proviruses are transcribed (Bieda et al., 2001). The transition of human melanoma cell lines from an adherent to a non-adherent growth phenotype is related to massive production of HERV-K-related viral-like particles and dependent on HERV-K expression, indicating that the activation of HERV-K expression may be a key element, though not necessarily the only one, contributing to morphological and functional cell changes during melanoma progression (Serafino et al., 2009).

### Other Phenomenon

Several other specific phenomena of HERV-K (HML-2) were identified. In ALS patients, a number of HERV-K (HML-2) protein variants other than full-length Env may potentially be expressed (Mayer et al., 2018; Garson et al., 2019). Splicing variants for *env* gene mRNA and transcripts from viral antisense strands were detected in prostate cancer cells (Agoni et al., 2013). A significant increase was detected in the frequency of the deletion of the 5′LTR-gag region of HERV-K111 in Caucasian patients with severe cutaneous T-cell lymphoma and/or Sezary syndrome when compared to healthy controls, indicating that pericentromeric instability is associated with more severe cutaneous T-cell lymphoma and/or Sezary syndrome (Kaplan et al., 2019).

## Conclusion

Correlation between the aberrant phenomenon of HERV-K (HML-2) and diseases has signaled HERV-K (HML-2) as a potential candidate for diagnosis or prognosis biomarkers. Although viral proteins were identified as onco-protein or autoantigen, the functional associations between HERV-K (HML-2) and diseases have not been completely clarified and further work is still needed to confirm HERV-K (HML-2) as causative factors. It is crucial to identify specific HERV loci associated with specific diseases, especially the polymorphic HERV-K (HML-2) loci, which may influence both the viral expression profile and the regulation to host-genes in different individuals. Although studies on the role of specific HERV-K (HML-2) loci were hampered by methodological limitations, with the emerging sequencing methods and bioinformatic tools, HERV-K (HML-2) biology is a compelling area for understanding causation and development of diseases.

## Author Contributions

BX and DK designed the structure of the article, performed the literature search, and wrote and edited the manuscript. LS reviewed and edited the manuscript before submission. All authors contributed to the article and approved the submitted version.

## Conflict of Interest

The authors declare that the research was conducted in the absence of any commercial or financial relationships that could be construed as a potential conflict of interest.
